# Sterilization of the Unfit*The demand for this article has been so great that I am compelled to reproduce it.—Daniel, Editor.

**Published:** 1911-02

**Authors:** G. Henri Bogart

**Affiliations:** Brookville, Indiana


THE
### TEXAS MEDICAL JOURNAL.
Established July, 1885

F. E. DANIEL, M. D., - - - - - Editor, Publisher and Proprietor

Published Monthly.—Subscription $1.00 a Year.

VOL. XXVI AUSTIN, FEBRUARY, 1911. No. 8

The publisher is not responsible for the views of the contributors.

### Original Articles.

### Sterilization of the Unfit.*

### THE LAW IN INDIANA, CONNECTICUT, CALIFORNIA, UTAH, OREGAN AND ONTARIO, CANADA.

BY DR. G. HENRI BOGART, BROOKVILLE, INDIANA.

The burden of degeneracy, feeble-mindedness and juvenile criminality, is increasing with multiplied ratios, the population by addition increments.

Every year the ratio of dependents, against which society must protect itself, increases out of proportion to the natural population development.

Modern sanitation has taken the weakling, whom natural selection would have sent to an early grave, and leads him past the perils of natural selection to maturity. With lack of balance, of will-power, of moral sways above animal desire, he proves a fertile breeder.

In the animal field wonderful results have been attained by selective breeding. The hazelsplitter hog has grown into the great storage batteries of food found in today's packing houses.

Burbank concentrated the evolution of the ages into days; the same course of nature that transformed a wild grass into the golden wheat was merely accelerated. But, in the man, castration and selective breeding are neither desirable nor possible.

Still, the fact remains that the imbecile and degenerate are laying an ever-swelling burden upon the shoulders of humanity.

*The demand for this article has been so great that I am compelled to reproduce it.—DANIEL, Editor.

These weaklings do not think, and, for momentary gratification, add a new member to the world—a member far better for himself and for all the world to have been unborn.

But it is ever darkest ere the dawn, and Omnipotent goodness, with His plan and purpose, best shown, from a materialistic standpoint, by evolution, can not allow His purpose to fail.

It is a fact that the forces of evil are usually more alert than are the elements of good, since the evil-doer expects immediate personal emolument, while the worker for good is too often allowed no reward, other than the altruistic one of consciousness of meritorious action.

When Dr. Harry C. Sharp was a student he learned, with horror, that much of the so-called "race suicide" results from the fact that many childless families are caused by a criminal operation. In the female, the Fallopian tube conveys the ovum from the ovaries to the uterus, where it is impregnated. Now, if this tube be cut, so that the ovum and spermatozoa can not meet, she can not reproduce her kind, yet her womanhood and her sexuality not only are not impaired, but are rather increased, since thwarted nature seeks to retrieve herself.

The young doctor experimented upon animals and found that though the operation was performed very early in life, the animal went on to full sexual maturity, only it was sterile. By analogy, he considered the male. There is a similar small tube, the vas deferens, which conveys the spermatozoa from the testicles to the seminal vesicles, where the semen is fertilized.

If now this tube be cut the testicles remain intact, sexual desire and accomplishment are unchanged, only that the subject is sterilized. All the horrors of unsexing and of castration are avoided. The operation is so slight that its explanation is hardly believable.

The vas deferens is a part of the spermatic cord and at the base of the penis, lies immediately beneath the skin.

The parts are rendered thoroughly aseptic, an incision three-eighths inch long is made through the skin, the cord is drawn up and the vas cut and tied at the vesicular end, to prevent a possible subsequent gonorrhea from extending back, and the wound is closed with a drop of collodion. The operation occupies but three minutes and is so slight that no anesthetic, either general or local, is used, and the patient climbs out of the chair and returns immediately to his ordinary duties.

The orchitic fluid and the spermatozoa are secreted as before, but instead of being partly dissipated by ejaculation are entirely reabsorbed by the lymphatics. This is the famous "elixir of life" of Brown-Sequard, and the tone and general well-being of the patient is immediately vastly improved.

Since first I made this operation public, in 1908, through the Medical Council, of Philadelphia, there has been an ever-widening class of physicians who are using it in confirmed masturbators to prevent the waste of vitality, and with tremendous benefits.

Negroes in confinement thus seek to relieve their abnormal sexuality more than whites, and Dr. Carrington, in charge of the State's prison of Virginia, has used it to control savage blacks, who had become insane through self-abuse, and every one of these violent creatures has become rational. The operation is not described in the standard works on surgery; indeed, it is practically an onward step, devised by Dr. Sharp. When that gentleman became physician to the Indiana Reformatory at Jeffersonville, Indiana, he began using it on those convicts who were neurasthenic, and the results were such that others voluntarily requested "the knife." The doctor reasoned that since the similar operation on the female was curtailing the breeding of the better classes through a hideous perversion, it were well to make the compensating balance by controlling the output of degeneracy. For ten years he operated by written consent of the convicts, and we sought, session after session, to get the matter made into law until on March 9, 1907, the Indiana Procreation law became a fact.

The great bar to an earlier passage of this law was the difficulty of showing the lay mind the difference between the horrors of castration and the beneficence of vasectomy.

We are sterilizing 25 per cent of our convicts, and there has not been an untoward result. True, the operation seems harsh; it is harsh, but so is the pulling of an aching tooth.

Allow me to quote the Jukes family, so much discussed by modern sociologists:

"Max, the progenitor, was born in New York, in 1720. He was a drunkard, who would not work. Of his descendants, 1206 have been identified as inmates of penal and charitable institutions, prior to 1874. Not one was ever elected to public office; not one ever served in army or navy, or contributed anything to public welfare. They cost society above $1,250,000. Three hundred and ten were in poorhouses, an average of over seven years each. One in four died in infancy; 440 are viciously diseased, 56 were notorious public prostitutes, 7 were convicted murderers, 60 were habitual thieves, 134 were habitual criminals."

Now had Dr. Sharp with his little knife encountered Max

Jukes' vas deferens, ere the above Pandora's box had been opened, what a saving of misery, sorrow, suffering and crime; a saving not only to general society, but to the wretched offenders, who would thus have been left unborn.

Now, it would have deprived Max of the pleasures of parentage, had he been sterilized, but what of all the degradation, the pain, the lost manhood and womanhood of his descendants. Apply the balance truly and dispassionately; which had been the better course?

Indiana was the first Commonwealth in all the world's history to seek to stop the flow of degeneracy, alienation and criminality by safe, humane and practicable nipping evil in the bud.

The law is as follows:

PREAMBLE.

WHEREAS, Heredity plays a most important part in the transmission of crime, idiocy and imbecility; therefore,

Be it enacted by the General Assembly of the State of Indiana, That, on and after the passage of this act, it shall be compulsory for each and every institution in the State entrusted with the care of confirmed criminals, idiots, imbeciles and rapists to appoint upon its staff two (2) skilled physicians and surgeons of recognized ability, whose duty it shall be in conjunction with the chief physician of the institution to examine the mental and physical condition of such inmates as may be recommended by the institutional physician and the board of managers. If in the judgment of this committee of experts and the board of managers procreation be inadvisable, and there is no probability of improvement of the mental condition of the said inmate, it shall be lawful for the surgeons to perform such operation for the prevention of procreation as shall be decided as safest and most effective.

But this operation shall not be performed save in such cases as have been pronounced unimprovable. Provided, that in no case shall the consultation fee be more than three dollars ($3) to each surgeon, to be paid out of the funds appropriated for the maintenance of such institution.

Approved March 9, 1907.

THE ONTARIO BILL.

Second Session, Twelfth Legislature, 10 Edward VII, 1910.
25th day of February, 1910. Mr. Godfrey.

No. 184. 1910.

BILL

An Act to prevent procreation by confirmed criminals, idiots, imbeciles and rapists.

WHEREAS, Heredity plays a most important part in the transmission of criminal instincts;

Therefore, His Majesty, by and with the advice and consent of the Legislative Assembly of the Province of Ontario, enacts as follows:

1. The governing body of every institution in which confirmed criminals, idiots, imbeciles and rapists may be confined, in addition to the regular institutional physician shall appoint two skilled surgeons of recognized ability whose duty it shall be in conjunction with the chief physician of the institution to examine such inmates as may be recommended by the chief physician as to their mental and physical condition.

2. If in the judgment of such surgeons procreation by any inmate is not advisable, and there is no probability of improving his mental condition, the surgeons may perform such operation on the inmate for the prevention of procreation as they shall deem most safe and effectual.

3. For every consultation as to the condition of an inmate of any such institution the surgeons concerned shall be entitled to a fee of not more than $3, to be payable out of the funds appropriated for the maintenance of the institution.

THE CONNECTICUT BILL.

[Substitute for House Bill No. 123.]

CHAPTER 209.

An Act concerning operations for the prevention of procreation.

*Be it enacted by the Senate and House of Representatives in General Assembly convened:*

SECTION 1. The directors of the State prison, and the superintendents of the State hospitals for the insane at Middletown and Norwich are hereby authorized and directed to appoint for each of said institutions respectively two skilled surgeons, who, in conjunction with the physician or surgeon in charge at each of said institutions, shall constitute a board, the duty of which shall be to examine such inmates of said institutions as are reported to them by the warden, superintendent, or the physician or surgeon in charge, to be persons by whom procreation would be inadvisable. Such board shall examine the physical and mental condition of such persons and their record and family history so far as the same can be ascertained, and if, in the judgment of a majority of said board, procreation by any such person would produce children with an inherited tendency to crime, insanity, feeble-mindedness, idiocy, or imbecility, and there is no probability that the condition of any such person so examined will improve to such an extent as to render procreation by any such person advisable, or if the physical or mental condition of any such person will be substantially improved thereby, then said board shall appoint one of its members to perform the operation of vasectomy or oophorectomy, as the case may be, upon such person. Such operation shall be performed in a safe and humane manner, and the board making such examination and the surgeon performing such operation shall receive from the State such compensation for services rendered as the warden of the State prison or the superintendent of either of such hospitals shall deem reasonable.

SEC. 2. Except as authorized by this act, every person who shall perform, encourage, assist in, or otherwise promote the performance of either of the operations described in Section 1 of this act, for the purpose of destroying the power to procreate the human species, or any person who shall knowingly permit either of such operations to be performed upon such person, unless the same shall be a medical necessity, shall be fined not more than one thousand dollars, or imprisoned in the State prison not more than five years, or both.

Approved August 12, 1909.

California and Utah have passed similar laws, but these three cover the benefits and defects sufficiently for the selection of a bill which shall cover all that experience has shown as best.

The Indiana law starts well, making the appointment of a surgical board mandatory, but when the actual operation is left to the caprice of a lay "board of managers," usually unsuccessful politicians who find in these boards a shelf sufficient to hold them in party line, it is weak.

By the words "in the State," the authorities have construed it to mean merely the main State institutions. The Ontario bill avoids these two errors.

In Indiana, because of the attitude of some of the boards, we have some institutions wherein the operation, though most needed, is not done.

The Connecticut bill is strong, in that its definition of the subjects for the operation is broad, in that it specifically includes both sexes, and in restricting private operation, but it is weak in confining the operation to the three institutions named, and it is not broad enough as concerns private practice. Epileptics should be included, as the operation is of unbelievable benefit to these patients.

We have here in Indiana a severe law against the marriage of degenerates and others unfit for procreation. We know that this class bear children, with or without marriage. Already handicapped in the battle, they drift aimlessly through life with no place or purpose, and eventually become public charges. Let such be sterilized and marry, and with home an object in the world be self-sustaining and self-respecting.

Allow me to quote two cases:

A lady, having a nice family, sustained a pelvic injury, so that at her last two pregnancies the children had to be dismembered to be delivered.

Another woman bore two children and then suffered thirteen abortions.

The two husbands came to us, and we operated on them, first having the conditions fully established. Both families are now happy.

The interest in this question is something phenomenal. I have published some thirty papers on this question, and my extra journals are loaned, even my private files being now loaned to a society in Kent, England.

I consider intelligent vasectomy, sterilization judiciously applied, as epochal, the greatest surgical advancement to a higher plane of humanity in the world's history.

The actual operation of vasectomy first appears in pastoral England, when sheep were wealth and wolves preyed. That the male lamb might retain his pugilistic powers, and defend the flock, his scrotum was liad over a block and bruised, the vas being occluded by this "tunking."

Many race suicide families follow a youthful gonorrhea which "went back," and by inflammatory action closed the vas or oviduct.

Among the nomadic Tartars, polyandria occurs; a woman who marries a man, wives his brothers. Hard bareback riding bruises the vas and sterilizes many, and as marriage contemplates procreation, conventionality alters to insure offspring. For the same reason, among the Guachos of the Pampas, male prostitution is prevalent among the elite, virile sailors and strangers being kidnapped and kept in not unwilling service for long periods.

It is a strange thing that astronomy began with the shepherds of the plains, and the milkmaids told Jenner of cowpox. Science finds much among the simple, and we are just getting great, good from evil, in turning intelligent vasectomy to the good, the incalculable good of humanity.

